# Dynamics of Mammalian Cell Infection by *Trypanosoma cruzi* trypomastigotes

**DOI:** 10.3389/fmicb.2020.559660

**Published:** 2020-10-02

**Authors:** Jorge A. Arias-del-Angel, Rebeca G. Manning-Cela, Moisés Santillán

**Affiliations:** ^1^Unidad Monterrey, Centro de Investigación y de Estudios Avanzados del Instituto Politécnico Nacional, Monterrey, Mexico; ^2^Departamento de Biomedicina Molecular, Centro de Investigación y de Estudios Avanzados del Instituto Politécnico Nacional, Mexico City, Mexico

**Keywords:** *Trypanosoma cruzi*, mammalian cell invasion, host-parasite interaction, infection kinetics, population dynamics model, time-delayed mathematical model

## Abstract

In a recent work we demonstrated that *Trypanosoma cruzi* trypomastigotes change their motility patterns in the presence of mammalian cells, that the extent of the changes depends on the cell line, and that this extent is positively correlated with the efficiency with which parasites invade the different cell lines. These results open the question of what cellular characteristics are relevant for parasite identification and invasion. In the present work, we tackled such question. We performed infection-kinetics experiments on various cell lines, and developed a mathematical model to simulate the experimental outcomes. An analysis of the cell-parasite mechanisms included in the model, together with the parameter values that allowed it to replicate the experimental results, suggests that a process related to the cell replication rate may strongly influence the parasite invasion efficiency, and the infection dynamics in general.

## 1. Introduction

Chagas disease was first described in 1909 by the Brazilian physician Carlos Chagas, after whom it is named. It is one of the neglected tropical diseases (NTDs) recognized by the World Health Organization (Rassi et al., 2012; Pérez-Molina and Molina, 2018). This disease, also known as American trypanosomiasis, is a tropical parasitic disease endemic to Latin America, that is caused by the protist *Trypanosoma cruzi*. Chagas disease is commonly spread to humans and other mammals by the blood-sucking *kissing bugs* of the subfamily *Triatominae*. The disease may also be spread through blood transfusion, organ transplantation, eating food contaminated with the parasites, and by vertical transmission (from a mother to her fetus). It is estimated that 8 million people, mostly in Mexico, Central America, and South America, have Chagas disease (WHO, 2020). Large-scale population movements have increased the areas where Chagas disease can be found, and these include many European countries, some African, Eastern Mediterranean and Western Pacific countries, Canada and the United States of America.

The interest in Chagas disease has increased over the last few years (Tanowitz et al., 1992; Paucar et al., 2016; Fonseca-Berzal et al., 2018; Lidani et al., 2019). Different aspects such as: development of new drugs, interaction of the parasite with the host immune system, parasite genomics, vaccine development, improved diagnosis methods, etc. are currently investigated by several groups worldwide. However, in spite of this increased interest, research on the parasite cell interaction from the standpoints of biophysics or non-linear dynamics is scarce.

It was shown than even when all *T. cruzi* stages are capable of establishing a cell infection *in vitro*, trypomastigote and amastigote stages exhibit a better infective performance than epimastigotes (Florencio-Martínez et al., 2010). However, in an *in vivo* scene, it has been demonstrated that only trypomastigotes and amastigotes are able to establish an infection; starting it within macrophages, smooth and striated muscle cells, as well as fibroblast (Andrade and Andrews, 2005). During this first cell-parasite interaction, *T. cruzi* amastigotes and trypomastigotes can employ different mechanism to get inside a cell (Burleigh and Woolsey, 2002; Andrade and Andrews, 2005). It has also been proposed that an interaction between a non-infected cell with an infected cell could encourage the infection of the first. This has been speculated as the movement of a parasite from the infected cell to the non-infected cell though the membrane of both cells, however this process has not been demonstrated yet.

In a recent work (Arias-del Angel et al., in press), we demonstrated that *T. cruzi* trypomastigotes of CL Brener strain change their motility patterns in the presence of *in vitro* cultured mammalian cells, albeit to a different extent depending on the cell line. Moreover, the extent of these changes is positively correlated with the efficiency with which trypomastigotes invade the studied cell lines. Although these results are quite suggestive, we were unable to pinpoint possible cell characteristics that influence parasite recognition and invasion. The present work is aimed at tackling this question, by means of an approach that combines experimental work and mathematical modeling.

The manuscript is organized as follows. In section 2, we present the materials and methods employed in our experiments. In section 3, we introduce a mathematical model for the interaction between parasites and mammalian cell cultures. In section 4, we present the results of infection-kinetics experiments with various mammalian cell lines, together with the corresponding numerical simulations. Finally, in section 5, we discuss the experimental and numerical results and derive the corresponding conclusions.

## 2. Materials and Methods

### 2.1. Cell Cultures

3T3 NIH embryonic mouse fibroblasts (ATCC® CRL-1658™), 3T3 Swiss-Albino (3T3-S) embryonic mouse fibroblasts (ATCC® CCL-92™), H9c2(2-1) rat myoblasts (ATCC® CRL-1446™), and Caco2 human colon epithelial (ATCC® HTB-37™) cells were grown in Dulbecco's Modified Eagle Medium (DMEM), supplemented with 10% fetal bovine serum (FBS) and 0.5% penicillin/streptomycin (100 μl/ml penicillin/streptomycin), at 37°C in an atmosphere of 5% CO_2_.

### 2.2. Parasite Cultures

Fluorescent epimastigotes from CL Brener strain were maintained in liver infusion tryptose (LIT) medium, supplemented with 10% fetal bovine serum (FBS), 0.5% penicillin (10,000 IU)/streptomycin (10,000 μg), and 1% hemin (5 mg/ml), at 28°C. These stable transfected parasites were obtained in a previous work by electroporation with pTREXn-GFP DNA (Florencio-Martínez et al., 2010). Cell-culture-derived trypomastigotes (CCDT) were obtained from supernatant of 3T3 NIH fibroblast monolayers infected with GFP-transfected parasites, as described below.

### 2.3. Cell Growth Kinetics

Inoculums of 2 × 10^4^ cells were seeded in well plates with DMEM + 10% FBS. To estimate the cell count in a given well, they were washed with PBS and dyed with 200 μl of Hoechst. The plates were then incubated in darkness at 37°C and 5% CO_2_ for at least 30 min. Afterwards, the wells were excited with a 405 nm laser diode, and 8 pictures were taken at random positions by means of a fluorescence microscope with a 10X objective. This configuration yielded images corresponding to well surfaces of 1.056 × 0.0845 mm. The obtained images were analyzed with a custom software implemented in MatLab. Basically, this software employs the bwdist and watershed functions to segment the parts of the image corresponding to nucleus. The nucleus in an image were then counted using the regionprops function.

### 2.4. Infection Kinetics

To obtain CCDTs, monolayers of NIH 3T3 cells grown to 50% confluence, were infected with 1 × 10^6^ mid-log-phase epimastigotes suspended in DMEM plus 2% FBS, on a final volume of 5 ml. The cells were washed 48 h after cell-parasites interaction with DMEM to remove non-adherent parasites, and fresh DMEM medium plus 2% FBS was added. This process was repeated every other day, following previous reports (Manning-Cela et al., 2001). The first released CCDTs were used for all experiments, because they contained very little amount of contaminating amastigotes. In infection kinetics, 1.95 × 10^5^ (1-3 parasites per cell) fluorescent CCDTs, obtained from the primary infection, were used to infect 3T3 NIH, 3T3-S, H9c2(2-1) and Caco2 cells grown over coverslips at 50% confluence. After 2 h of cell-parasite interaction, the cultures were washed with DMEM to remove non-adherent parasites, and the culture medium (DMEM plus 2% FBS) was renewed every 48 h during 18 days. Coverslips were recovered at different times (0.75, 2, 3, 5, 7, 9, 11, 13, and 18 days post initial-interaction) and then washed with 1X PBS, fixed with formaldehyde at 3.7% for 20 min at room temperature, washed again with 1X PBS, and finally mounted and stained with Vectashield with DAPI (*Vector*, Cat. 1,500). The samples were analyzed with a LEICA confocal microscope, with a 40X oil-immersion objective (NO 24) and the fluorescence images captured and analyzed using the software MatLab. As the trypomastigote stage was used to initiate the infections, a host cell was considered infected if at least one amastigote was observed inside a cell (Lentini et al., 2017). The percentage of infected cells was calculated by comparing the number of cells containing parasites to the total number of cells.

## 3. Model Development

**Figure 1:**
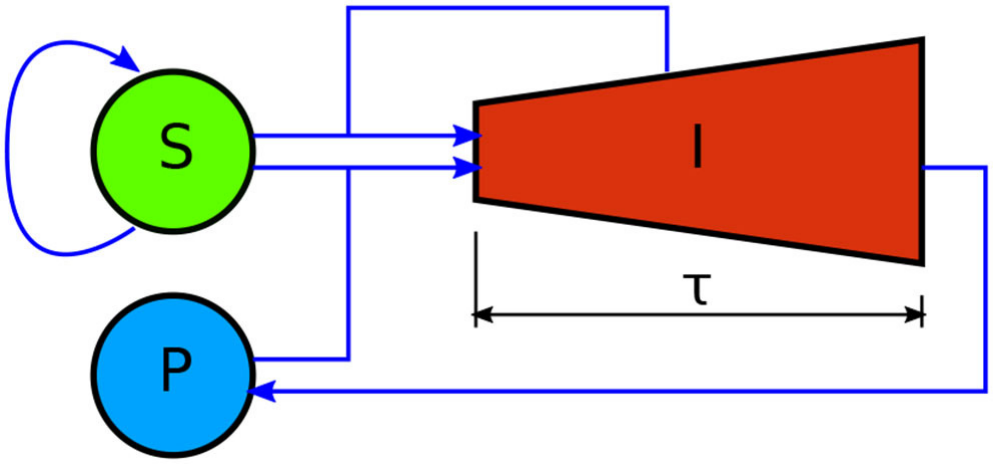
Schematic representation of the mathematical model here developed to account for the dynamics of mammalian cell infection by *T. cruzi* trypomastigotes. *S*, *I*, and *P*, respectively stand for the amount of susceptible mammalian cells, infected mammalian cells, and parasites. Blue arrows denote interactions between compartments. Susceptible cells self-replicate. They get infected *via* interactions with infected cells and parasites. Unlike the other model variables, infected cells have an age structure, indicated by the shape of the corresponding compartment. They undergo an incubation period of length τ, after which they are lysed and liberate numerous new parasites to the medium. Infected cells also self-replicate. This is represented by the increasing height of compartment *I*.

In this section, we develop a model for the dynamics of mammalian cell infection by *T. cruzi* trypomastigotes—see Figure 1 for a schematic representation. The model is inspired on the classical epidemiological model first introduced by Kermack and McKendrick (1991). It considers three different populations: susceptible cells (*S*), infected cells (*I*), and free parasites (*P*), whose evolution is governed by the following set of delay differential equations:

(1) $$\begin{array}{l} \frac{dS}{dt}=r_{S}S\left(1-\frac{S+I}{K}\right)-\left[\beta SP+\gamma SI\right], \end{array}$$

(2) $$\begin{array}{l} \frac{dI}{dt}=r_{I}I\left(1-\frac{S+I}{K}\right)+\left[\beta SP+\gamma SI\right]\\ \;-\left[\beta S_{\tau }P_{\tau }+\gamma S_{\tau }I_{\tau }\right]\exp\left[r_{I}\int_{t-\tau }^{t}\left(1-\frac{S\left(t^{\prime }\right)+I\left(t^{\prime }\right)}{K}\right)dt^{\prime }\right], \end{array}$$

(3) $$\begin{array}{l} \frac{dP}{dt}=\delta \left[\beta S_{\tau }P_{\tau }+\gamma S_{\tau }I_{\tau }\right]\exp\left[r_{I}\int_{t-\tau }^{t}\left(1-\frac{S\left(t^{\prime }\right)+I\left(t^{\prime }\right)}{K}\right)dt^{\prime }\right]\\ \;-\beta SP. \end{array}$$

The model assumes that the susceptible cells can get infected when they get in contact with either parasites or infected cells; that infected cells undergo an incubation period of length τ, after which they are lysed and liberate several parasites to the environment; and that susceptible and infected cells can replicate according to logistic equations, while they compete for medium resources. Equations (1)–(3) are balance equations, and the meaning of the corresponding right-hand-side (rhs) terms is as follows. The first rhs terms in Equations (1) and (2) respectively account for the growth rates of susceptible and infected cells. Regarding the parameters in these terms, *r*_*S*_ and *r*_*I*_ are the intrinsic growth rate constants of susceptible and infected cells, while K is the environment carrying capacity. The second rhs terms in Equations (1) and (2) stand for the rate of infection of susceptible cells due to interactions with parasites or infected cells. These terms assume mass-action-law kinetics. β is the rate constant for the infection of susceptible cells by parasites, and γ is the rate constant for the infection of susceptible cells when they get in contact with infected ones. The last term in Equation (3) accounts for the fact that, when a trypomastigote invades a cell, it stops being a free parasite. The third rhs term in Equation (2) takes into consideration that the number of infected cells that die at time *t*, equals the number of them that got infected at time *t* − τ (*X*_τ_ denotes *X*(*t* − τ)), times a factor of the form $\exp\left(\int_{t-\tau }^{t}\mu \left(t^{\prime }\right)dt^{\prime }\right)$—with μ(*t*′) the replication rate of infected cells—which accounts for the replication of infected cells in the interval [*t* − τ, *t*] (see the Appendix A1 for a formal derivation). Finally, the first rhs term in Equation (3) accounts for the fact that each time an infected cell is lysed, it liberates δ trypomastigotes to the environment. Observe that Equations (1)–(3) lack death rate terms for parasites and cells (other than the lysis of infected cells). The reasons for this are that: in the case of mammalian cells, such death rates are intrinsically incorporated into the logistic growth rates; whereas in the case of parasites, our experimental protocol contemplates the removal of the culture medium (and thus of the majority of free parasites) every other day, and parasite death is negligible in such period of time.

## 4. Results

**Figure 2:**
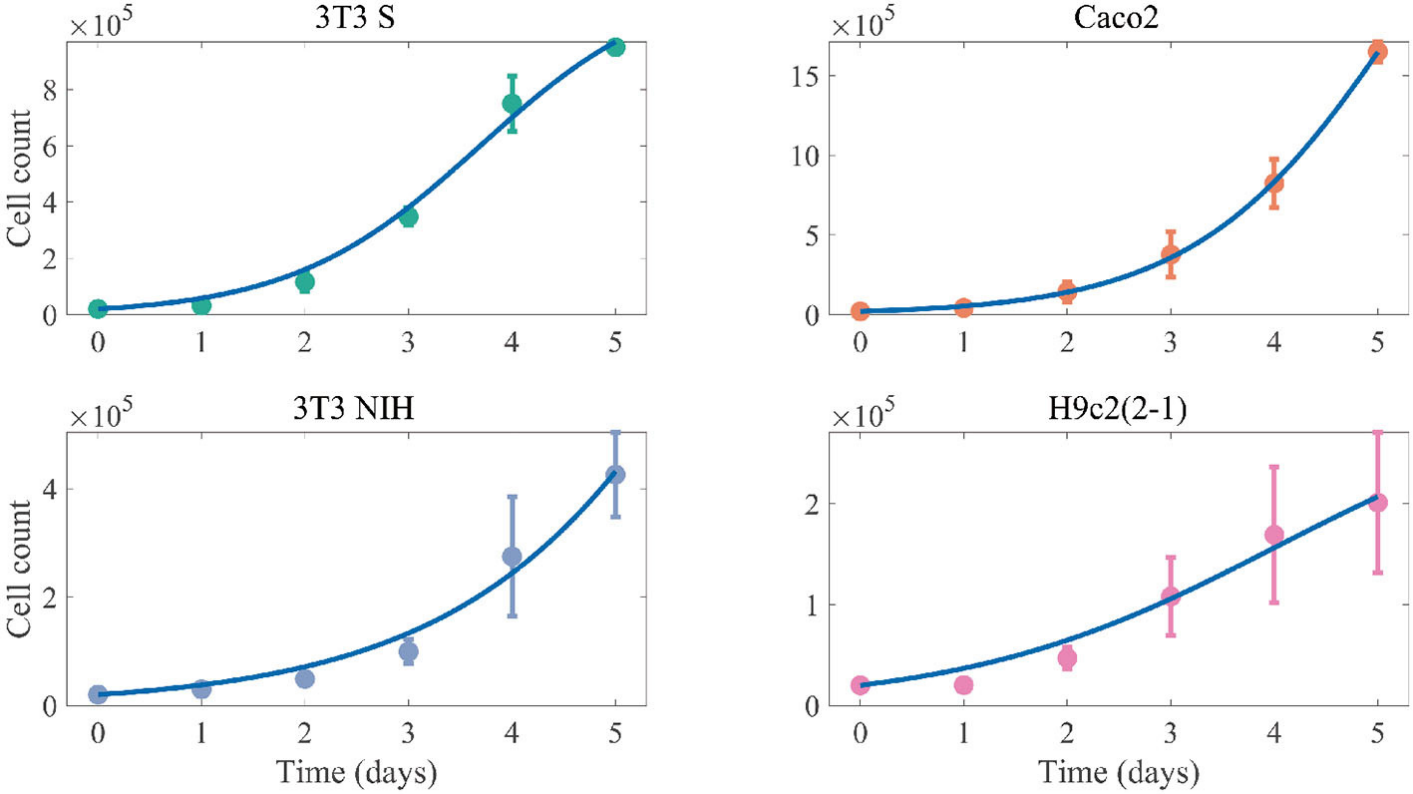
Cell-count time evolution in cultures of different mammalian cell lines. The dots represent experimental values (averaged over 3 independent experiments), while the error bars denote standard deviations. The solid lines correspond to the best fitting logistic functions.

We started by characterizing the growth kinetics of the different cell lines here studied. Following the methodology in the section 2, we cultured the 3T3 S, Caco2, 3T3 NIH, and H9c2(2-1) cell lines, and measured the time evolution of the corresponding cell counts. The obtained results are presented in Figure 2. Notice that the cell growth curves can be fitted by the solution of Equation 1, without the infection terms, which corresponds to the following logistic function:

$$S=\frac{K}{1+\left(\frac{K-S_{0}}{S_{0}}\right)e^{-r_{S}t}}.$$

We performed the fitting by means of MatLab's algorithm lsqcurvefit. The best fitting curves are shown in Figure 2 for all cell lines, and the corresponding best-fitting parameter values are tabulated in Table 1. The resulting values for *r*_*S*_ and *K* can be respectively interpreted as the intrinsic growth rates of susceptible cells and the corresponding carrying capacities.

**Table 1:** Logistic-function best fitting parameter values for all analyzed cell lines.

**Cell line**	***r*_*S*_ (×10^-3^*h*^-1^)**	**K (×10^6^ cells)**
3T3 S	45.83	1.2
Caco2	41.25	3.88
3T3 NIH	27.92	2.78
H9c2(2-1)	27.92	0.31

We followed the infection kinetics of the 3T3 S, Caco2, 3T3 NIH, and H9c2(2-1) cells, using the methodology described in the section 2. In short, we added 1-3 parasites per cell to ~50%-confluence cell cultures, and let them interact for 2 h before exchanging the culture medium. Afterwards, we washed and changed the culture medium (repeating this procedure every other day), and measured the fraction of infected cells at different times. Since we employed GFP-transfected parasites, and given that amastigote is the parasite obligated intracellular stage, a recently infected cell could be identified because there is at least one amastigote (a spherical green spot) nearby the cell nucleus (Lentini et al., 2017). The fraction of infected cells was then calculated as the ratio of cells with amastigotes (or trypomastigotes for advanced infections) within to the total cell count in a picture. Regarding the number of parasites per infected cell, we estimated this parameter by means of a custom program in MatLab when only amastigotes were present. In the case of the cells with trypomastigotes, we could not perform the estimation due to the geometrical irregularity of this parasite stage. The average parasite count at 18 h and 7 days post intractions are tabulated in Table 2.

**Table 2:** Average parasite count per infected cell at different times post interaction (TPI).

**Cell line**	**17 h. TPI**	**7 days TPI**
3T3 S	1	12.83
Caco2	0	13.00
3T3 NIH	1.2	26.66
H9c2(2-1)	1.14	36.95

**Figure 3:**
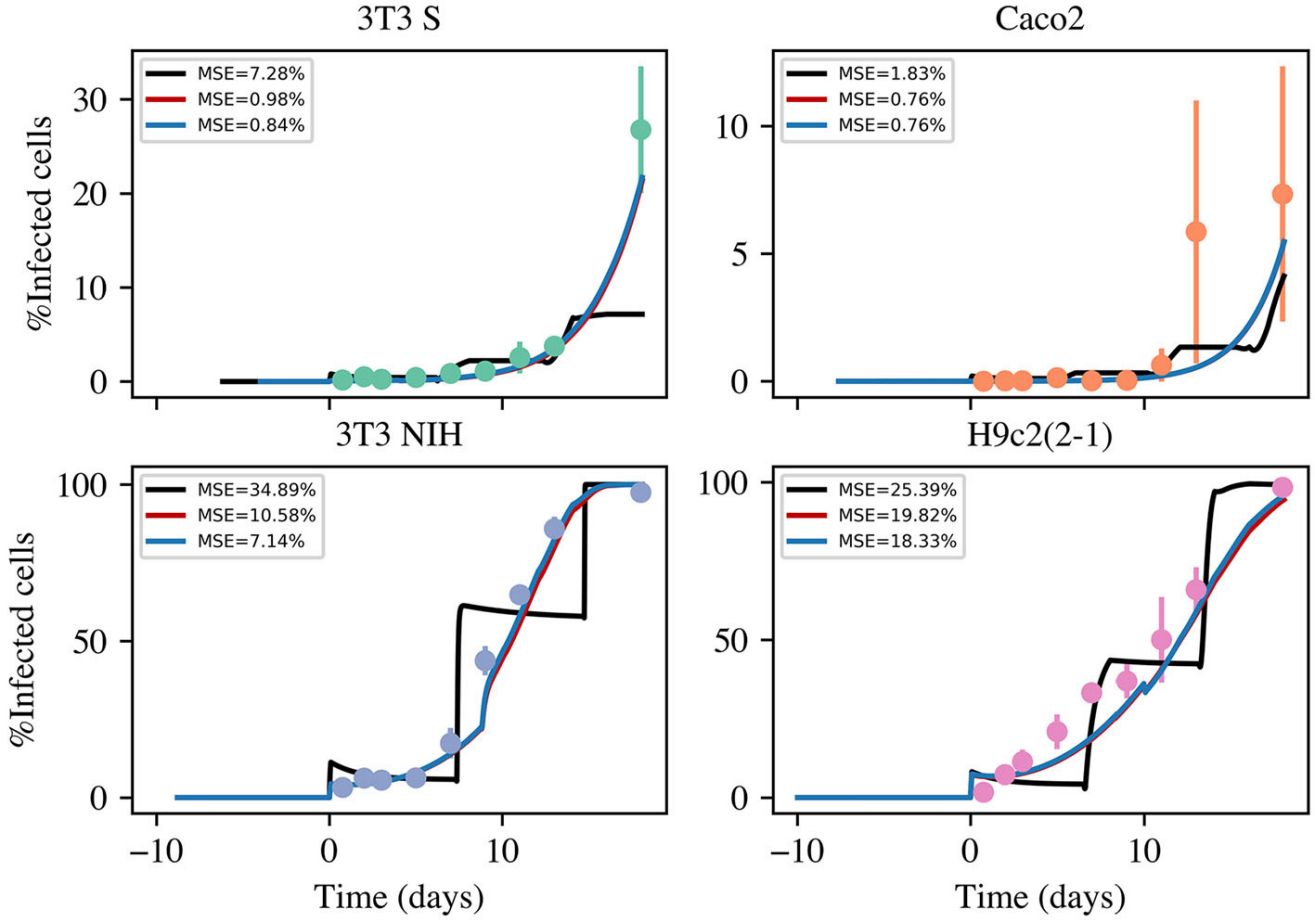
Time evolution of the fraction of infected cells in infection kinetics experiments with different mammalian cell lines. The dots represent experimental values (averaged over 3 independent experiments), while the error bars denote standard deviations. The solid lines correspond to the best fitting curves obtained from numerical solutions of three model versions. The color code is as follows: black, basic model; red, intermediate model; and blue, most complete model. The acronym MSE in the figure legends stands for mean standard error.

The results concerning the evolution of the fraction of infected cells are shown in Figure 3. Interestingly, the employed cell lines follow very different infection kinetics, despite the infectious agent being the same in all cases. To better make sense of the infection-kinetics results, we performed simulations using the formerly introduced mathematical model. To this end, we implemented in MatLab the following numerical protocol, which involves numerically solving the model equations by means of the Euler's algorithm (with Δ*t* = 0.01 h):

1. This step simulates the process of culturing the mammalian cells until they get a ~50% confluence. Notice that, when *I* = *P* = 0, the model equation system reduces to the logistic model for the susceptible compartment. Taking this into account, we solved the resulting logistic ordinary differential equation, with *S*(*t*_0_) = 0.01 *K*, for a time long enough so that *S*(*t*_*f*_) = *K*/2, with *t*_*f*_ the final simulation time. Then, we set *t* ← *t* − *t*_*f*_ so the next step starts at time zero.
2. To simulate the interaction between cells and parasites, we numerically solved the model system of delay differential equations for 2 h, using the solution of the previous step as initial condition for *S*. The initial condition for *I* was set to zero for all *t* ≤ 0. The initial condition for *P* was zero for *t* < 0, and to *P*_0_ for *t* = 0, with $P_{0}=1.95\times 10^{5}$ (which correspond to 1-3 parasites per cell at a confluence of 50%).
3. The next simulation step consisted in numerically solving the model delay differential equation system for 48 h (i.e., the initial and final simulation times were *t* = 2 h and *t* = 50 h), with the accumulated solution for *S*, *I*, and *P* as initial condition, except that *P* was set to zero at *t* = 2 h to simulate the culture medium replacement.
4. Step 3 was iterated, increasing the simulation time by 48 h in each iteration, until the total simulation time reached the duration of the experiment (18 days).

We attempted to use Matlab's dde23 built method to numerically solve the model equations. However, it was not possible to adapt it to the specific needs of our simulations. More specifically, in the experimental setup, we let parasites interact with ~50%-confluence cell cultures for 2 h, and then renewed the cell culture medium (and thus removed free swimming parasites) every other day, to guarantee cell viability. Thus, to mimic such experiments, we needed to numerically solve the model equations in time intervals of different lengths, and use the accumulated partial solution as the initial condition for the next step. Since the format of dde23's returned solution is incompatible with these requirements, we decided to program our own numeric algorithm.

In principle, we can simulate the infection-kinetics experiments by running the above described numerical protocol, provided we have accurate parameter estimations. As explained above, we were able to experimentally obtain estimates for *r*_*S*_ and *K*. For the rest of the parameters (β, γ, δ, τ, and *r*_*I*_), we do not have good estimates. Thus, we performed the Inverse Analysis by inferring these parameter's values using Bayesian statistics (Colin et al., 2013). We assumed Gaussian priors for all parameters. The corresponding mean values were selected by empirically finding parameter values that rendered a good fit. The standard deviations were set equal to 1/10 times the mean values. We experimented with wider priors, but we did not obtain satisfactory fits. Let $f_{i}^{\left(e\right)}$ be the fraction of infected cells experimentally measured at times $t_{i}^{\left(e\right)}$, and let *f*^(*s*)^(*t*) = *I*(*t*)/(*I*(*t*) + *S*(*t*)) be the fraction of infected cells predicted by the simulation with a given set of parameter values. From this, the likelihood function we used for the Bayesian inference analysis is as follows:

$$\exp\left(-\frac{1}{2}\frac{\sum_{i}{\left(f_{i}^{\left(e\right)}-f^{\left(s\right)}\left(t_{i}\right)\right)}^{2}}{\sigma^{2}}\right),$$

with σ = 2.5. To obtain Monte Carlo samples from the posterior distribution, we made use of a t-walk Markov chain Monte Carlo (MCMC) method (Christen and Fox, 2010). This is a self-adjusting MCMC algorithm and the resulting sampler is efficient in most cases for this low dimensional problem.

Using the above described method, we considered three different version models. One (the simplest) which only considers infections *via* cell-parasite interactions and replication of non-infected cells. An intermediate model that also takes into account cell-to-cell infections. And, finally, the most complete model, which contemplates cell-to-cell infections and replication of infected cells. The results are summarized in Figure 3. The priors for all parameters and the histograms of the corresponding posterior samples are shown in Appendix A2 for all model versions. The model solutions plotted in Figure 3 were computed with the mean values of the corresponding posterior samples. Observe that the most basic model is incapable of reproducing the experimental results. Instead of yielding monotonically growing curves, it returns stair-like responses. The reason is that, when parasites are removed every other day, the infection stops abruptly and one has to wait until new parasites are released for cell infection to continue. When cell-to-cell infections are taken into consideration, the infection process can continue, even when parasites are removed from the culture medium. This is the reason why the intermediate model is able to reproduce the experimental results. Finally, when proliferation of infected cells is incorporated into the model, the fitting errors are reduced a little, but not significantly. In conclusion, our results suggest that cell-to-cell infection plays an important role during the kinetics of *T. cruzi* infections, while replication of infected cells is not so important from an infection-dynamics perspective. From the above discussion, we decided to use the intermediate model version from now on. The mean values and standard deviations (obtained from the posterior samples) for all the parameters in the intermediate model are tabulated in Table 3.

**Table 3:** Intermediate-model best fitting parameter values for all analyzed cell lines.

**Cell line**	**β **(*h*^-1^*parasites*^-1^)****	**γ **(*h*^-1^*cells*^-1^)****	**δ (parasites)**	**τ (h)**
3T3 S	2.38 × 10^−9^ ± 3.4 × 10^−12^	1.08 × 10^−8^ ± 8.04 × 10^−12^	6.80 ± 0.15	95.9 ± 0.11
Caco2	1.05 × 10^−10^ ± 2.65 × 10^−12^	0.48 × 10^−8^ ± 3.22 × 10^−11^	9.18 ± 0.79	182 ± 7.54
3T3 NIH	1.40 × 10^−7^ ± 3.56 × 10^−10^	0.49 × 10^−8^ ± 5.33 × 10^−12^	5.21 ± 0.03	212 ± 0.80
H9c2(2-1)	2.00 × 10^−7^ ± 5.12 × 10^−9^	4.08 × 10^−8^ ± 1.14 × 10^−10^	1.93 ± 0.03	240 ± 0.08

## 5. Discussion and Concluding Remarks

In this work we have performed *in vitro* infection-kinetics experiments with *T. cruzi* CL Brener strain and different mammalian cell lines. The variability of the obtained results is noteworthy. Intriguingly, the infections progresses are considerably more slowly in 3T3 S than on 3T3 NIH cells, in spite of both being fibroblasts. Whereas the totality of 3T3 NIH cells are infected by day 15, only about one third of 3T3 S have been infected at day 18. This difference on the infectious behavior of *T. cruzi* trypomastigotes has already been reported, indicating that the infectious performance can change, not only among parasite strains, but also according to the infected cell line (Melo and Brener, 1978; Bertelli and Brener, 1980; Vargas-Zambrano et al., 2013; Zingales, 2018). It is also interesting that Caco2 cells are very hard to infect (only about 10 percent of the cultured cells have been infected by day 18), despite they are derived from colon, and this is one of the more infected organs by CL Brener *T. cruzi* strain (Zingales et al., 2012). We attempted to extend the experiments for longer periods of time in the case of 3T3 S and Caco2 cells. However, it was not possible because the cells detached from the glass substrate. Since, *in vivo* infection experiments have shown that *T. cruzi* parasites show some preference to infect cardiac and digestive-system tissues, it could be expected that Caco2 cells were more efficiently infected that other cell lines. However, we obtained the opposite result. Interestingly, our experimental results agree with those of Martello et al. (2013), who found that, when infected with trypomastigotes (Brazillian strain), the percentage of infected Caco2 cells never exceeded 0.15% after 11 days of the initial interaction. We believe this paradoxical result has to do with the fact that Caco2 cells are derived from cancerous tissue, and some of the genetic modifications caused by this disease have made them resistant to infection by *T. cruzi* trypomastigotes.

To help us make sense of the above discussed results, we developed a mathematical model for the infection dynamics. The results of the model simulations are shown in Figure 3, where we can appreciate how the simulations fit the experimental results. To get these fits, we had to incorporate into the model the assumption that susceptible cells can get infected when they interact with infected cells. We also considered the possibility that infected cells can replicate, but it resulted unnecessary. In this regard, our modeling results suggest that cell-to-cell transmission of the parasite plays a non-negligible role in the infection dynamics.

A few facts about the parameter values in Table 3 deserve further discussion:

- The obtained τ values correspond to incubation times in the range from 4 to 10 days, which agree with experimental observations.
- The value of parameter γ resulted to be at least twice as large for the H9c2(2-1) than for any other cell line. This makes sense if we consider that H9c2(2-1) cells are myoblasts, and thus cell contacts are more prominent than in other cell lines, even at medium confluences.
- Intriguingly, the estimated values of δ (average number of parasites released per lysed infected cell) are consistently smaller than the measured average count of amastigotes per infected cell (Table 2). We do not have a proper explanation for this discrepancy, but believe that it may be to the fact that parasite-cell interactions are modeled *via* the mass action law, which implicitly assumes that the system is well mixed. In our experimental setup, the system not only cannot be considered well mixed, because the cells are immobile, but the distribution of mammalian cells is 2-dimensional, while that of parasites is 3-dimensional. This topology implies that only those parasites in the vicinity of the monolayer can eventually infect the cells in it. Thus, the mass action law approximation would only be valid for a small region above the cell monolayer. Since in our experiments, most of the parasites released by infected cells swim away, this could explain the low δ values. Interestingly, Sibona et al. (2005) fitted a different population model to *T. cruzi* populations in mice. They found a relatively small number of parasites emerging from a cell burst (between 2.2 and 14), depending on mice and parasite strain. These values are not too far from the ones we obtained here for the *in vitro* experiments.

**Figure 4:**
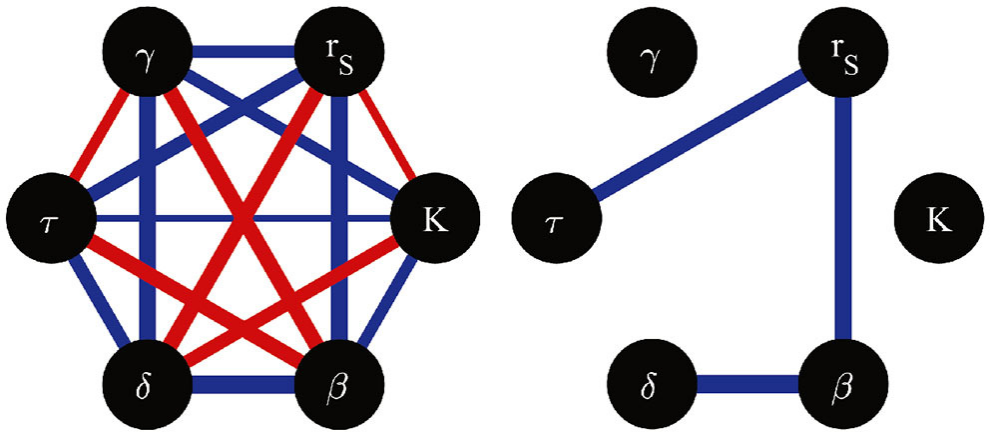
Network of correlation coefficients between pairs of model parameters, computed from the parameter values estimated for the four studied cell lines. Red (blue) edges indicate positive (negative) correlations. The absolute value of the correlation coefficient is represented by the corresponding edge width. All possible edges are illustrated in the left hand side graph. The right hand side graph only shows the edges corresponding to correlation coefficients with absolute vales larger than 0.9.

One can notice the existence of some correlations among the best-fitting parameter values in Table 3. For instance, parameter *r*_*S*_, seems to be negatively correlated with β. To explore this in a more quantitative way, we computed the Pearson correlation coefficient between all pairs of parameters, considering the four studied cell lines. The results are illustrated in Figure 4. Observe that only a few correlations have absolute values larger than 0.9, among them, that between *r*_*S*_ and β. Although it is impossible to demonstrate a cause-effect relation from variable correlations, the notion (derived from the correlation network in Figure 4) that a mechanism related to the cell replication rate, *r*_*S*_, may strongly influence the efficiency of infection, β, is very suggestive. Indeed, it could help us understand why Caco2 cells are so resistant to *T. cruzi* infection (being derived from cancerous tissue, they have a large proliferation rate). If a functional relation could be identified between the rate of proliferation and the rate of infection of a given cell line, it could have potential biomedical implications.

## Data Availability Statement

The raw data supporting the conclusions of this article will be made available by the authors, without undue reservation.

## Author Contributions

JA-d-A performed research, contributed analytic tools, analyzed data, and contributed to writing the manuscript. RM-C and MS designed research, contributed analytic tools, performed research, analyzed data, and wrote the manuscript. All authors reviewed the manuscript.

## Conflict of Interest

The authors declare that the research was conducted in the absence of any commercial or financial relationships that could be construed as a potential conflict of interest.

## Figures and Tables

**Figure 5:**
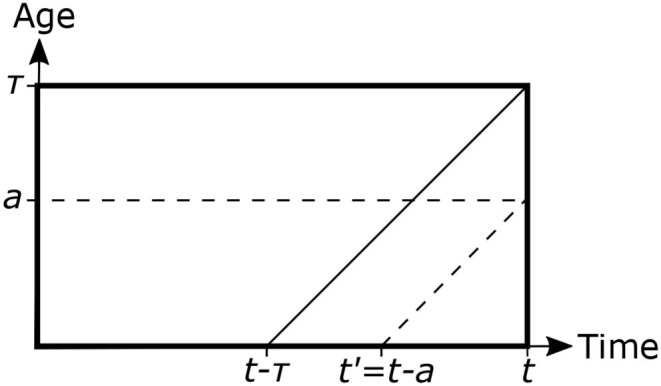
Scheme representing the age structure of a population of infected mammalian cells. τ represents the age at which infected cells are lysed and liberated the incubated parasites to the environment. Inclined lines are the characteristics of the partial differential equation governing the system dynamics, and indicate how the solution at any (*t, a*) point can be traced back to the *a* = 0 axis.

## Appendix

### A1. Delayed Dynamics of Infected Cells

Consider a population of mammalian cells that got infected at different times. Let *a* represent the time since a given cell got infected. For simplicity let us call *a* the age of the infected cell. Following M'Kendrick (1925), the age structure of this population can be represented by *i*(*t, a*)*da*, which represents the number of cells that have ages in the interval [*a, a* + *da*] at time *t*. The time evolution of *i*(*t, a*) is governed by the following partial differential equation:

$$\begin{array}{l} \frac{\partial i}{\partial t}=-\frac{\partial i}{\partial a}+\mu \left(t\right)i. \end{array}$$

The first term in the right hand side of the former equation accounts for the decrease of variable *i* due to the aging of cells, while the second term represents cell proliferation. If we take into account that *a* is not independent from *t* but obeys the relation *a* = *t* − *t*_0_, with *t*_0_ the time of infection, then: ∂*i*/∂*t* + ∂*i*/∂*a* = *di*/*dt*, which leads to the following differential equation:

$$\begin{array}{l} \frac{di}{dt}=\mu \left(t\right)i. \end{array}$$

According to the method of characteristics, the solution to this differential equation is:

$$\begin{array}{l} i\left(t,a\left(t\right)\right)=i\left(t_{0},a\left(t_{0}\right)\right)\exp\left(\int_{t_{0}}^{t}\mu \left(t^{\prime }\right)dt^{\prime }\right). \end{array}$$

Recalling that *a* = *t* − *t*_0_, the above expression can be rewritten as:

$$\begin{array}{l} i\left(t,a\right)=\sigma \left(t-a\right)\exp\left(\int_{t-a}^{t}\mu \left(t^{\prime }\right)dt^{\prime }\right), \end{array}$$

where σ(*t*) = *i*(*t*, 0) is the flux of newly infected cells at time *t*. Let $I\left(t\right)=\int_{0}^{\tau }i\left(t,a\right)da$ be the total number of infected cells at time *t* (τ is the age at which infected cells complete maturation and are lysed, liberating several new parasites to the environment). Substitution of the last equation allows us to translate the integral from the axis of age to that of time as follows—see Figure 5:

$$I\left(t\right)=\int_{t-\tau }^{t}\sigma \left(t^{\prime }\right)\exp\left(\int_{t^{\prime }}^{t}\mu \left(t^{\prime \prime }\right)dt^{\prime \prime }\right)dt^{\prime }.$$

Let us now differentiate *I*(*t*) with respect to time

$$\begin{array}{l} \frac{dI}{dt}=\frac{d}{dt}\int_{t-\tau }^{t}\sigma \left(t^{\prime }\right)\exp\left(\int_{t^{\prime }}^{t}\mu \left(t^{\prime \prime }\right)dt^{\prime \prime }\right)dt^{\prime }. \end{array}$$

By making use of the fundamental theorem of calculus we obtain:

$$\begin{array}{l} \frac{dI}{dt}=\mu \left(t\right)I+\sigma \left(t\right)-\sigma \left(t-\tau \right)\exp\left(\int_{t-\tau }^{t}\mu \left(t^{\prime }\right)dt^{\prime }\right). \end{array}$$

This last equation can be interpreted as a balance equation. The rate of change of infected cells is given by appearance of new cells due to proliferation, plus the flux of newly infected cells, minus the flux of cells undergoing lysis (which equals the flux of newly infected cells at time *t* − τ, times an exponential term that account for cell proliferation in the interval [*t* − τ, *t*]).

### A2. Parameter Priors and Posteriors for All Version Models

Priors are shown with solid lines, while the histograms resulting from posterior samples are shown with bar plots. The model versions are coded as follows: A), B) y C) corresponds to the simplest, intermediate, and most complete model, respectively.
